# Adoption rates of electronic health records in Turkish Hospitals and the relation with hospital sizes

**DOI:** 10.1186/s12913-020-05767-5

**Published:** 2020-10-21

**Authors:** Ilker Kose, John Rayner, Suayip Birinci, Mustafa Mahir Ulgu, Ismayil Yilmaz, Seyma Guner, HIMSS Analytics Team, Suna Kirdag Mahir, Kubra Aycil, Beytiye O. Elmas, Esra Volkan, Zeynep Altinbas, Gizem Gencyurek, MoH Team, Esra Zehir, Berrin Gundogdu, Mert Ozcan, Ceyhan Vardar, Behcet Altinli, Jale Sungur Hasancebi

**Affiliations:** 1 Department of Health System Engineering, Istanbul Medipol University, 34810 Istanbul, Turkey; 2 HIMSS Analytics for Europe and Latin America, Huddersfield, UK; 3 Ministry of Health, 06800 Ankara, Turkey; 4 Istanbul Medipol University, 34810 Istanbul, Turkey

**Keywords:** Electronic health records; meaningful use; CPOE; PACS, eMAR, CDSS, EMRAM, HIMSS

## Abstract

**Background:**

Nation-wide adoption of electronic health records (EHRs) in hospitals has become a Turkish policy priority in recognition of their benefits in maintaining the overall quality of clinical care. The electronic medical record maturity model (EMRAM) is a widely used survey tool developed by the Healthcare Information and Management Systems Society (HIMSS) to measure the rate of adoption of EHR functions in a hospital or a secondary care setting. Turkey completed many standardizations and infrastructural improvement initiatives in the health information technology (IT) domain during the first phase of the Health Transformation Program between 2003 and 2017. Like the United States of America (USA), the Turkish Ministry of Health (MoH) applied a bottom-up approach to adopting EHRs in state hospitals. This study aims to measure adoption rates and levels of EHR use in state hospitals in Turkey and investigate any relationship between adoption and use and hospital size.

**Methods:**

EMRAM surveys were completed by 600 (68.9%) state hospitals in Turkey between 2014 and 2017. The availability and prevalence of medical information systems and EHR functions and their use were measured. The association between hospital size and the availability/prevalence of EHR functions was also calculated.

**Results:**

We found that 63.1% of all hospitals in Turkey have at least basic EHR functions, and 36% have comprehensive EHR functions, which compares favourably to the results of Korean hospitals in 2017, but unfavorably to the results of US hospitals in 2015 and 2017. Our findings suggest that smaller hospitals are better at adopting certain EHR functions than larger hospitals.

**Conclusion:**

Measuring the overall adoption rates of EHR functions is an emerging approach and a beneficial tool for the strategic management of countries. This study is the first one covering all state hospitals in a country using EMRAM. The bottom-up approach to adopting EHR in state hospitals that was successful in the USA has also been found to be successful in Turkey. The results are used by the Turkish MoH to disseminate the nation-wide benefits of EHR functions.

## Background

Electronic health records (EHRs), as defined by the Turkish Ministry of Health (MoH), is any information recorded, stored, transmitted, accessed, correlated, and processed by using electronic systems related to past, present and future physical and mental health condition or diseases of individuals [1]. This information repository, when used in a meaningful manner, keeps all of the records that are useful, effective, ethical, and easily accessible within regulated boundaries [2, 3]. The key functions contained in EHRs are computerized physician order entries (CPOE) [4–7], closed-loop medication administration records (CLMA) [8–12], clinical decision support systems (CDSS) [13–15], picture archiving and communication systems (PACS) [16], and electronic medication administration records (eMAR) [9, 17, 18]. Hospital information systems (HIS) combine these functions with additional modules essential to clinical and administrative processes. Common modules include patient administration systems (PAS) capable of recording the identification and demographic data of patients [19]. Despite varying content and structure due to the local health insurance system, electronic medical billing (EMB) systems are another crucial HIS component integrated with EHRs and PAS [20].

### Overall electronic health record capabilities of countries

There are only a few studies evaluating the national adoption level of EHR functions in hospitals. One of the first and widely cited studies was conducted in 2009 by Jha et al., which surveyed the availability of 24 EHR functions in US hospitals [21]. Results were classified according to whether the hospitals had basic or comprehensive EHR functions. Basic EHR functions indicate that clinical documentation, CPOE, CDSS, and laboratory and imaging results are limited to one clinic, while comprehensive EHR functions indicate availability in all clinics of the hospital. The study showed that only 1.5% of US hospitals had comprehensive EHR functions, and 7.6% had basic EHR functions. A subsequent study in 2011, which was also conducted by Jha et al., indicated that the total percentage of US hospitals having at least basic EHR functions had increased to 15.1% [22]. Adler-Milstein et al. conducted more recent studies in 2014 [23], 2015 [24], and 2017 [25]. These studies showed that the proportion of US hospitals having comprehensive EHR functions was 25.5% in 2014, 34.1% in 2015, and 39.1% in 2017. Similarly, the proportion of hospitals having at least basic EHR functions was 58.9% in 2014, 75.2% in 2015, and 80.5% in 2017.

Studies focusing on Korean hospitals are also noteworthy. The first study was conducted by Park et al. in 2005 [26]. Researchers used a survey designed by Ash et al. [27], which focused more on CPOE than other EHR functions. This study showed that although 80.3% of hospitals have CPOE forms, only 9% have complete EHR systems. Two subsequent studies conducted in Korean hospitals used the Jha et al. [21] survey, which allowed them to compare their results to the Jha et al. US hospital results. A 2012 study by Yoon et al. [28] showed that the percentage of Korean hospitals having at least basic EHR functions was 37.2%, which was higher than the proportion of hospitals in the USA (15.1%). The most recent study published in 2017 by Kim et al. [29] showed that the percentage of Korean hospitals having at least basic EHR functions had increased to 58.1%. Still, this figure was lower than the proportion of US hospitals with at least basic EHR functions (80.5%) for the same year. The rapid increase in the adoption rate of EHRs in US hospitals may be attributed to the financial and political support provided by the HITECH Act (2009).

Another notable study was published in 2014 by Shu et al. [30]. This cross-sectional study measured the rate of EHR adoption in tertiary hospitals in China. The authors conducted a national survey entitled the Model of EHR Grading (MEG), which gives hospitals a rating between Stage 0 and 7 based on their adoption of 37 EHR functions. This study showed that, among 848 hospitals, 30.7% were Stage 0, 12.0% were Stage 1, 31.7% were Stage 2, 22.2% were Stage 3, 2.7% were Stage 4, 0.6% were Stage 5, 0.1% were Stage 6, and 0% were Stage 7. Other studies performed in Greece [31] and Saudi Arabia [32] were not survey-based, but rather thematic reviews focused on providing insights for policymakers.

Kanakubo and Kharrazi write one of the most recent studies comparing country-wide EHR adoption level in 2019. This study compares two countries by gathering data set from the Healthcare Information and Management Systems Society (HIMSS) for the USA, and from the Ministry of Health, Labour and Welfare (MHLW) for Japan for the years of 2008, 2011, and 2014. The results of this study showed that while the USA and Japan have similar status in 2008 and 2011, the USA became better than Japan in adopting EHR for small, medium, and large hospitals in 2014. On the other hand, the government hospitals in Japan are better than US hospitals for all 3 years [33].

Approaches to encouraging the adoption of EHRs in hospitals is a critical point for policymakers. United Kingdom (UK) and US strategies to stimulate the adoption of EHR functions are of particular interest. Aziz et al., in 2014, asserted that many lessons should be learned from US achievements with a bottom-up approach and criticized the UK’s top-down decision-making [34]. They attributed the US success to the three distinct stages of the implementation-strategy and stated that the UK strategy lacked clearly defined milestones Owen et al. responded to this criticism of the UK approach by highlighting the 100% adoption rate of EHRs at the primary care level when US rates remained considerably lower [35]. In a recent study published in 2018, Wilson and Khansa also compared the EHR systems of the UK and the USA. They noted that the top-down strategy brought early success to the UK with general practitioners, but noted that this strategy was not successful when the UK tried to bring EHRs to hospitals because of the complexity of processes among stakeholders. They suggested that even though the USA had the most extensive private healthcare system in the world, which might be more challenging to control, their bottom-up approach seemed more successful than the top-down approach of the UK, which had the most extensive public healthcare system in the world [36].

### Electronic health record capability in Turkey

The MoH in Turkey launched the Health Transformation Program in 2003 [37] and finalized the first phase in 2017. Many milestones were achieved with this national healthcare reform program that related to the use of information and technology standards such as the National Health Data Dictionary (NHDD) [38], the Health Coding Reference Server [39], the International Classification of Diseases, 10th Revision (ICD-10) [40], Health Level Seven International (HL7) [41, 42], National Health Tariffs [43], and other systems including the Family Physician Information System [44], the National Health Information System [38], the Central Physician Booking System [45], the Central Claims Management System [46], a teleradiology system [47], and a Personal Health Records system [48, 49]. As an essential part of the national health information technology infrastructure, the MoH initiated a pilot national e-prescription system in June 2012, which became mandatory as of January 2013. Although the target is set as 95%, the adoption rate of the e-prescription system was 87% as of September 2018 [50].

Although there are many nation-wide standards and applications in Turkey, there had been no measurement of adoption rates for new systems within healthcare facilities (i.e., hospitals, health centers, etc.). The MoH of Turkey decided to conduct a study measuring the overall adoption rates of EHR functions within state hospitals. One of the objectives of the MoH Strategic Plan for 2013–2017 was to study the “digital hospital” concept and disseminate it across all state hospitals [51]. In line with this strategic objective, the MoH signed a collaborative agreement with the Healthcare Information and Management Systems Society (HIMSS) in 2013 [52] and decided on using EMRAM. EMRAM is a powerful survey which is applied by more than 25 countries including some EU countries, Turkey, China, Russia, Saudi Arabia, etc., and very common in the USA so that there are 2039 and 285 validated hospitals against the requirements of Stage 6 and Stage 7 correspondingly in the USA only [53]. Besides Turkey, Portugal is also considering to use EMRAM as a country-wide measure for digital transformation [54], and Canada applied a customized version of it for community-based physicians [55]. Since it is an EHR adoption model, EMRAM does not focus on human and organizational capabilities but the technological functionality of the hospital [56].

The Turkish MoH’s role as a policymaker allowed hospitals to conduct relevant studies within the period determined by the Strategic Plan. As such, Turkey also applied a US-style bottom-up approach to encouraging the adoption of EHRs in state hospitals.

### Electronic medical health record adaptation model

The electronic medical health record adaptation model (EMRAM) [57] developed by HIMSS provides algorithms to assess inpatient services in acute hospitals based on their EHR capabilities and, like all other models [58–60] created by HIMSS, has eight stages from 0 to 7. EMRAM is first developed in 2005 and enhanced by HIMSS to meet the technological progress of the overall digitalization of hospitals [61]. The assessment criteria for hospitals in our study are shown in Table 1 [57]. The EMRAM survey is currently used in over 50 countries worldwide, has been used over 60,000 times to assess digital maturity and nearly 3000 times to validate hospitals at either Stage 6 or Stage 7. HIMSS suspects that approximately 830 m people have been impacted by healthcare providers using one or more maturity models.

**Table 1:** HIMSS EMRAM Requirements (as of January 1, 2018)

Stage	Cumulative Capabilities
Stage 7	Complete Electronic Medical Record (EMR); Continuity of Care Document transactions to share data; Data warehousing; Data Continuity with Emergency Department, Ambulatory, and OP
Stage 6 *(Comprehensive EHR functions)*	Physician documentation (structured templates); Full Clinical Decision Support System (CDSS) (variance & compliance); Full Remote-PACS
Stage 5	Closed-Loop Medication Administration (CLMA)
Stage 4	Computerized Physician Order Entry (CPOE); CDSS (clinical protocols)
Stage 3 *(basic EHR functions)*	Nursing/clinical documentation (flow sheets); CDSS (error checking); PACS available outside of Radiology
Stage 2	Clinical Data Repository (CDR); Controlled Medical Vocabulary; CDSS, May have Document Imaging; Health Information Exchange (HIE) capable
Stage 1	Ancillaries - Lab, Rad, Pharmacy - All Installed
Stage 0	All Three Ancillaries not Installed

The EMRAM is a simple, well-evaluated model that requires users to assess levels of compliance with a straight forward “yes/no” or “Present/Absent” responses. If the response is positive, which means the relevant application or function is available, occasionally, the subject is asked to set the percentage of compliance or usage coverage (i.e., departmental, hospital-wide, etc.).

## Methods

This study utilizes the EMRAM survey, which includes five dimensions: Software Applications (SA), Software Usage (SU), Electronic Ordering (EO), Image Management Systems (IMS), and Medication Safety (MS). While the SA dimension investigates the availability of software and EHR functions, the SU dimension focused on the prevalence of them. The EO dimension focuses on e-order usage in drug and non-drug orders, and CDSS usage in e-orders. The IMS dimension investigates the archiving and retrieving the clinical images. Finally, the MS focuses on CLMA records through the hospital.

To compare this study with previous studies conducted with US and Korean hospitals, EMRAM requirements are aligned with the 24 functions of the survey developed by Jha et al. [21] so that Stage 3, 4 and 5 of EMRAM indicates that the hospital has basic EHR functions, and Stage 6 indicates that the hospital has comprehensive EHR functions. Kanakubo and Kharrazi do a similar alignment in 2019 to benchmark between the USA and Japan [33].

The survey studies took place between 2014 and 2017. Sixteen regional workshops were organized in the same period, with 2716 participants, including hospital managers, from 870 distinct state hospitals. This study aimed to cover all 870 state hospitals [62] in Turkey, and all hospitals are invited to fulfill the survey. Surveys that were of inadequate quality were excluded from this study. In cases where the same hospital submitted more than one survey having the same score, only the most recent survey was analyzed.

We preferred using the “adoption of EHR” phrase instead of “use of EHR” or “adoption and use of EHR” in our study for two reasons: 1) The EMRAM is the abbreviation for the electronic medical record “adoption” model. The adoption, here, means the availability and use of EHR functions within the hospital. 2) While some respectful authors preferred using “Use of EHR” [21, 63, 64] in the meaning of “the availability and use of some EHR functions” in their papers, some other authors [24, 25, 28, 65, 66] used “EHR adoption” in the same meaning as we preferred.

IBM SPSS version 23 was used to perform statistical analyses. The Chi-Square test was conducted to analyze associations or differences between the categorical variables, such as hospital size and the adoption rate of EHR functions. The threshold for significance was set at α = 0.05.

## Results

A total of 889 surveys were collected from 870 hospitals. Of the 889 surveys collected, 204 were excluded as duplicate submissions. The number of distinct hospitals that completed surveys was 685 (78.7%). Of these, 85 surveys were excluded because of poor quality data and severe inconsistencies. Overall, 600 verified and acceptable surveys (68.9%) were analyzed in this study. The distribution of the hospitals and their size, type, and level of healthcare are shown in Table 2. All seven of Turkey’s regions, 97.5% of Turkey’s 79 provinces, and 68.9% of all Turkish hospitals were represented in the sample. Small hospitals represent 49.2%, medium-sized hospitals represent 34.2%, and Large hospitals represent 16.7% of the participating hospitals. All hospitals participating in the study are state hospitals (88.5%), while 11,5% are training hospitals. Of participating hospitals, 81.5% were secondary hospitals, 9% were tertiary hospitals, and 9.5% were specialized hospitals.

**Table 2:** Basic characteristics of responding hospitals

Characteristic	Total Number in Turkey	Participating Hospitals	Participating Percentage (%)	Percentage in Sample (%)
Region	7	7	100	100
Province	81	79	97.5	100
Hospital	870	600	68.9	100
Size				
Small (0–99 beds)	531	295	55.5	49.2
Medium (100–399 beds)	232	205	88.3	34.2
Large (≥400 beds)	117	100	85.4	16.7
Teaching Status				
Public Hospital	775	531	68.5	88.5
Training Hospital	95	69	72.6	11.5
Hospital Type^a^				
Secondary Hospital	682	489	71.2	81.5
Tertiary Hospital	95	54	56.8	9
Branch Hospital	93	57	61.2	9.5

^a^ *All hospitals in the sample are public (state) hospitals*

**Fig. 1:**
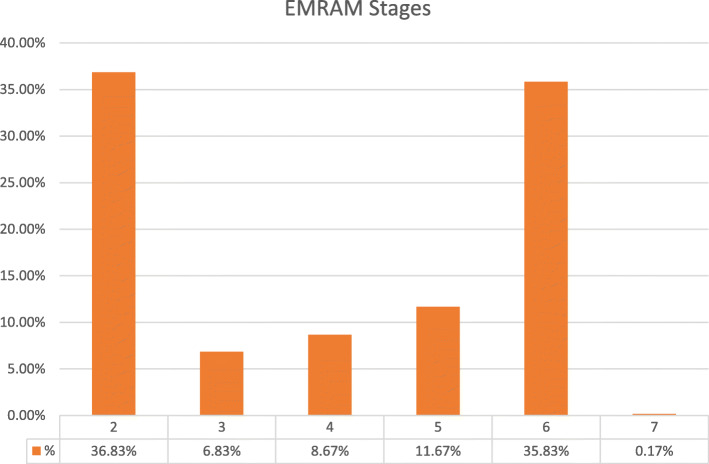
Distributions of hospitals at each EMRAM Stage

The distribution of the number of hospitals in each EMRAM stage is shown in Fig. 1. It is remarkable that most hospitals (72.66%) are either at Stage 2 or 6. The barrier requirements of Stage 3, such as having PACS, eMAR, and adopting nursing documents, appear to be a threshold, as once hospitals achieve Stage 3, the remaining technologies seem to be adopted relatively easily and adoption levels increase steadily. If we consider these results in terms of the categories developed by Jha et al. in 2009, 36% of hospitals have Comprehensive EHR functions, and 63% have Basic EHR functions.

### Availability of applications and electronic health record functions

This section provides results regarding the availability of information systems and EHR functions in hospitals.

#### Hospital information systems, laboratory information systems, and patient administration systems

The availability results of HIS, Laboratory Information Systems (LIS), and PAS are listed in Table 3 according to hospital size. The results show that 100% of hospitals have a HIS suite. Similarly, 94% of hospitals have PAS, and 93% of hospitals have LIS integrated with their HIS. It is also evident that hospital size does is not positively associated with having HIS, LIS, and PAS. The survey investigates whether the application is live only in the relevant department such as laboratory, etc. or live in the entire hospital. Thus such the tables have two corresponding columns as “Live” and “Live -Hospital-wide”.

**Table 3:** Availability of HIS, LIS and PAS

Applications	Hospital Size	Live	Live - hospital-wide	Live – departmental	Not Automated	Missing	Total
EMR / Hospital Information System (Suite)	Large (> = 400 beds)	100 (100%)	0 (0.0%)	0 (0.0%)	0 (0.0%)	0	**100**
	Medium (100–399 beds)	205 (100%)	0 (0.0%)	0 (0.0%)	0 (0.0%)	0	**205**
	Small (6–99 beds)	294 (99.6%)	1 (0.34%)	0 (0.0%)	0 (0.0%)	0	**295**
	**Percentage**	**99.8%**	**0.2%**	**0.0%**	**0.0%**	**0.0%**	**100%**
	**Total**	**599** **(99.8%)**	**1** **(0.16%)**	**0** (0.0%)	**0** (0.0%)	**0**	**600**
Patient Administration System	Large (> = 400 beds)	1 (1%)	98 (98%)	0 (0.0%)	1 (1%)	0	**100**
	Medium (100–399 beds)	2 (0.99%)	189 (93.56%)	3 (1.48%)	8 (3.96%)	3	**205**
	Small (6–99 beds)	5 (1.70%)	269 (91.80%)	9 (3.07%)	10 (3.41%)	2	**295**
	**Percentage**	**1.3%**	**92.7%**	**2.0%**	**3.2%**	**0.8%**	**100%**
	**Total**	**8** **(1.34%)**	**556** **(93.44%)**	**12** **(2.01%)**	**19** **(3.19%)**	**5**	**600**
Laboratory Information System	Large (> = 400 beds)	41 (41%)	57 (57%)	2 (2%)	0 (0.0%)	0	**100**
	Medium (100–399 beds)	43 (21.07%)	153 (75%)	7 (3.43%)	1 (0.49%)	1	**205**
	Small (6–99 beds)	7 (2.37%)	257 (87.11%)	29 (9.83%)	2 (0.67%)	0	**295**
	**Percentage**	**15.2%**	**77.8%**	**6.3%**	**0.5%**	**0.2%**	**100%**
	**Total**	**91** **(15.19%)**	**467** **(77.96%)**	**38** **(6.34%)**	**3** **(0.5%)**	**1**	**600**

#### Clinical documents and computerized Physician order entry

Results related to clinical documentation (CDR) are provided in Table 4, according to hospital size. The results show that 98.6% of hospitals have a CDR, and 79.7% of hospitals have a hospital-wide CDR. CDR is one of the requirements of EMRAM Stage 2 and Physician and nursing documents, as requirements of EMRAM Stage 3, have very similar availability percentages across hospital sizes. While 86.2% of hospitals have physician documents, nursing documents are available in 84.8% of all hospitals. Those high percentages may be explained by the Turkish national healthcare quality standards (SKS), which have required a clinical document infrastructure since 2009. CPOE, on the other hand, is not functional in 13.8% of hospitals. The size of the hospital has no significant relationship to the availability of clinical documents and CPOE systems.

**Table 4:** Availability of clinical documents and CPOE systems

Applications	Hospital Size	Live	Live - hospital-wide	Live - departmental	Not Automated	Missing	p
Clinical Data Repository (CDR)	Large (> = 400 beds)	42 (42%)	56 (56%)	1 (1%)	1 (1%)	0	*p* < 0.001
	Medium (100–399 beds)	44 (21.5%)	154 (75.1%)	4 (2%)	3 (1.5%)	0	
	Small (6–99 beds)	7 (2.4%)	268 (91.2%)	16 (5.4%)	3 (1%)	1	
	**Percentage**	**15.5%**	**79.7%**	**3.5%**	**1.2%**	**0.2%**	
	**Total**	**93**	**478**	**21**	**7**	**1**	
Nursing Documentation	Large (> = 400 beds)	24 (24%)	55 (55%)	2 (2%)	19 (19%)	0	*p* < 0.001
	Medium (100–399 beds)	25 (12.3%)	131 (64.5%)	10 (4.9%)	37 (18.2%)	2	
	Small (6–99 beds)	6 (2%)	230 (78.2%)	26 (8.8%)	32 (10.9%)	1	
	**Percentage**	**9.2%**	**69.3%**	**6.3%**	**14.7%**	**0.5%**	
	**Total**	**55**	**416**	**38**	**88**	**3**	
Physician Documentation	Large (> = 400 beds)	28 (28%)	53 (53%)	3 (3%)	16 (16%)	0	*p* < 0.001
	Medium (100–399 beds)	32 (15.8%)	128 (63.1%)	11 (5.4%)	32 (15.8%)	2	
	Small (6–99 beds)	6 (2.0%)	234 (79.6%)	22 (7.5%)	32 (10.9%)	1	
	**Percentage**	**11.0%**	**69.2%**	**6.0%**	**13.3%**	**0.5%**	
	**Total**	**66**	**415**	**36**	**80**	**3**	
CPOE	Large (> = 400 beds)	25 (25%)	55 (55%)	2 (2%)	18 (18%)	0	*p* < 0.001
	Medium (100–399 beds)	31 (15.1%)	132 (64.4%)	8 (3.9%)	34 (16.6%)	0	
	Small (6–99 beds)	3 (1%)	234 (79.6%)	26 (8.8%)	31 (10.5%)	1	
	**Percentage**	**9.8%**	**70.2%**	**6.0%**	**13.8%**	**0.2%**	
	**Total**	**59**	**421**	**36**	**83**	**1**	

#### Medication administration

Pharmacy and medication administration are essential functions of Hospital Information Systems. As depicted in Table 5, the surveys indicate that 99.5% of all hospitals have a pharmacy management system, even though three (0.5%) of them receive this service from an external vendor. Medications administered to the patient are recorded at the point of service in 66% of the hospitals, but not in the other 29.2%. The high availability of pharmacy management systems can be explained by MoH regulations addressing stock management and efficiency criteria for state hospital pharmacies that have been in place since 2013. Despite the high availability of information systems, the lack of medication application recording implies that information systems are still more focused on institutional purposes like billing than on clinical services.

**Table 5:** Availability of medication administration systems

Applications	Hospital Size	Live	Live –hospital-wide	Live – departmental	Installation in Process	Not Automated	ESP attached	Missing	Total
Pharmacy Management System	Large (> = 400 beds)	41 (41%)	55 (55%)	4 (4%)	0 (0%)	0 (0%)	0 (0%)	0	**100**
	Medium (100–399 beds)	43 (20.97%)	144 (70.24%)	15 (7.3%)	0 (0%)	2 (0.97%)	1 (0.48%)	0	**205**
	Small (6–99 beds)	8 (2.71%)	247 (83.72)	37 (12.54%)	0 (0%)	1 (0.33%)	2 (0.67%)	0	**295**
	**Percentage**	**15.3%**	**74.3%**	**9.3%**	**0.0%**	**0.5%**	**0.5%**	**0.0%**	**100%**
	**Total**	**92**	**446**	**56**	**0**	**3**	**3**	**0**	**600**
Electronic Medication Administration Record	Large (> = 400 beds)	30 (30%)	46 (46%)	4 (4%)	1 (1%)	19 (19%)	0 (0%)	0	**100**
	Medium (100–399 beds)	29 (14.28%)	110 (54.18%)	3 (1.47%)	0 (0%)	61 (30.04%)	0 (0%)	2	**205**
	Small (6–99 beds)	5 (1.70%)	176 (60.06%)	17 (5.80%)	0 (0%)	95 (32.42%)	0 (0%)	2	**295**
	**Percentage**	**10.7%**	**55.3%**	**4.0%**	**0.2%**	**29.2%**	**0.0%**	**0.7%**	**100%**
	**Total**	**64**	**332**	**24**	**1**	**175**	**0**	**4**	**600**

#### Image management

As depicted in Table 6, survey results show that 89% of hospitals have a PACS system; but the PACS systems in 14.5% of hospitals are not integrated with the hospital’s HIS and may only be available to the radiology department. Only 9.5% of hospitals have a dictation and speech recognition system to help radiologists write their reports more efficiently, so technology adoption has not yet penetrated the reporting process.

**Table 6:** Availability of image management systems

Applications	Hospital Size	Live	Live - hospital-wide	Live - departmental	Installation in Process	Service Not Provided	Not Automated	Missing	Total
Dictation with Speech Recognition	Large (> = 400 beds)	1 (1%)	6 (6%)	6 (6%)	0 (0.0%)	0 (0.0%)	46 (46%)	41	**100**
	Medium (100–399 beds)	1 (0.64%)	15 (9.61%)	5 (3.20%)	0 (0.0%)	0 (0.0%)	135 (86.53%)	49	**205**
	Small (6–99 beds)	1 (0.35%)	16 (5.65%)	6 (2.12%)	0 (0.0%)	0 (0.0%)	260 (91.87%)	12	**295**
	**Percentage**	**0.5%**	**6.2%**	**2.8%**	**0.0%**	**0.0%**	**73.5%**	**17.0%**	**100%**
	**Total**	**3**	**37**	**17**	**0**	**0**	**441**	**102**	**600**
Radiology - Central PACS	Large (> = 400 beds)	31 (31%)	56 (56%)	1 (1%)	1 (1%)	1 (1%)	9 (9%)	1	**100**
	Medium (100–399 beds)	34 (16.58)	137 (66.8%)	24 (11.70%)	0 (0.0%)	1 (0.48%)	9 (4.39%)	0	**205**
	Small (6–99 beds)	4 (1.36%)	185 (63.13%)	62 (21.16%)	0 (0.0%)	1 (0.34%)	41 (13.99%)	2	**295**
	**Percentage**	**11.5%**	**63.0%**	**14.5%**	**0.2%**	**0.5%**	**9.8%**	**0.5%**	**100%**
	**Total**	**69**	**378**	**87**	**1**	**3**	**59**	**3**	**600**

### Usage of applications and electronic health record functions

This section presents results regarding the usage and dissemination of information systems and EHR functions in hospitals.

#### Clinical documents and computerized Physician order entry

As shown in Table 4, Physician Documents (PD) systems are available in 84.6% of hospitals, but we found significant variations in the levels of PD systems (Table 7). A vast majority of hospitals (79.4%) have electronic medical record systems, and 74.5% of hospitals are using PDs in at least 50% of the hospital. The ratio-generating discrete data obtained from the PDs are also other critical indicators that represent the capacity to extract information from the medical record. We found that 75.2% of the hospitals with PDs are using discrete data in PDs in at least 50% of the hospital. The regulations of the Turkish MoH can also explain this high percentage of discrete data usage. The MoH accredits HIS vendors annually according to their capability to submit relevant datasets to the MoH as outlined in the NHDD since 2015 [38].

**Table 7:** Prevalence of physician documents

SW Usage	Hospital Size	51–100%	1–50%	Not Applicable	Total	Missing	P
What percent of all current medical records are electronic (incl. Digital/scanned data)?	Large (> = 400 beds)	59 (59%)	0 (0.0%)	0 (0.0%)	**100**	41	0.017*
	Medium (100–399 beds)	152 (74.14%)	9 (4.39%)	0 (0.0%)	**205**	44	
	Small (6–99 beds)	265 (89.83%)	29 (9.83%)	0 (0.0%)	**295**	1	
	**Percentage**	**79.3%**	**6.3%**	**0.0%**	**100.0%**	**14.3%**	
	**Total**	476	38	0	**600**	86	
What percent of Physician Documentation generates discrete (computer-readable) data?	Large (> = 400 beds)	71 (71%)	6 (6%)	16 (16%)	**100**	7	**0.321**
	Medium (100–399 beds)	150 (73.17%)	13 (6.34%)	34 (16.58%)	**205**	8	
	Small (6–99 beds)	230 (77.96%)	24 (82.75%)	32 (93.60%)	**295**	9	
	**Percentage**	**75.2%**	**7.2%**	**13.7%**	**100.0%**	**4.0%**	
	**Total**	451	43	**82**	**600**	**24**	
What percent of physicians use the Physician Documentation system?	Large (> = 400 beds)	73 (73%)	6 (6%)	16 (16%)	**100**	5	**0.826**
	Medium (100–399 beds)	149 (72.68%)	16 (7.80%)	34 (16.58%)	**205**	6	
	Small (6–99 beds)	225 (92.8%)	26 (8.81%)	41 (43.15%)	**295**	3	
	**Percentage**	**74.5%**	**8.0%**	**15.2%**	**100.0%**	**2.3%**	
	**Total**	447	48	**91**	**600**	**14**	

*p < 0.05

Electronic ordering also has a similar prevalence. The usage of CPOE is 72.5% for medication and 62.7% for non-medication orders, respectively. However, orders for nurses in inpatient care facilities have a slightly higher proportion of 79%. Table 9 shows that the percentage of CPOE usage for inpatient medication orders is 66.6 and 70.5% for non-medication orders in more than 50% of the hospital.

Although there is no significant relationship between hospital size and the use of electronic ordering (Table 8), the prevalence of electronic ordering has a significant relationship with hospital size (Table 9). The results show that small hospitals are better than medium-sized and larger hospitals in adopting electronic ordering capabilities. Verbal orders are not allowed according to regulations applicable to Turkish state hospitals. However, it seems that managers can enforce this rule better in smaller hospitals.

**Table 8:** Availability of electronic ordering

Questions	Hospital Size	Yes***	No***	Not Applicable***	Missing***	P
Electronic ordering for medication	Large (> = 400 beds)	54 (90.0%)	5 (8.3%)	1 (1.7%)	40	0.004**
	Medium (100–399 beds)	140 (85.9%)	11 (6.7%)	12 (7.4%)	42	
	Small (6–99 beds)	241	41	5	8	
	**Percentage**	**72.5%**	**9.5%**	**3.0%**	**15.0%**	
	**Total**	**435**	**57**	**18**	**90**	
Electronic ordering for non-medication	Large (> = 400 beds)	41 (97.6%)	1 (2.4%)	0 (0.0%)	58	0.016*
	Medium (100–399 beds)	90 (98.9%)	1 (1.1%)	0 (0.0%)	114	
	Small (6–99 beds)	245 (91.4%)	23 (8.6%)	0 (0.0%)	27	
	**Percentage**	**62.7%**	**4.2%**	**0.0%**	**33.2%**	
	**Total**	**376**	**25**	**0**	**199**	
Electronic ordering for nursing and/or physician services	Large (> = 400 beds)	75 (76.5%)	5 (5.1%)	18 (18.4%)	2	0.001**
	Medium (100–399 beds)	160 (78.4%)	17 (8.3%)	27 (13.2%)	1	
	Small (6–99 beds)	239 (81.3%)	37 (12.6%)	18 (6.1%)	1	
	**Percentage**	**79.0%**	**9.8%**	**10.5%**	**0.7%**	
	**Total**	**474**	**59**	**63**	**4**	

*p < 0.05, **p < 0.01 *** The availability of the information system such as PACS, and dictation system is investigated, as in Table 6, using the following set of selections: “Live; Live - hospital-wide; Live - departmental; Installation in Process; Service Not Provided; Not Automated; Missing”. It is just because such an information system can be applied at the departmental level. On the other hand, the availability of administration or application of EHR functions, such as e-order, clinical documentation, etc. is investigated with the following set of selections: “Yes; No; Not Applicable; Missing” since they are either exist or not. If it is reasonable, the prevalence of some of those functions is separately investigated, as in Tables 9 and 14

**Table 9:** Prevalence of electronic ordering

Questions	Hospital Size	100% (all)	76–100%	51–75%	26–50%	1–25%	Not Applicable	Missing	P
What % of all inpatient medication orders are processed electronically?	Large (> = 400 beds)	0	48 (81.35%)	3 (5.08%)	1 (1.69%)	2 (3.38%)	5 (8.47%)	41	**0.856**
	Medium (100–399 beds)	0	123 (81.45%)	9 (5.96%)	2 (1.32%)	4 (2.64%)	13 (8.6%)	54	
	Small (6–99 beds)	0	203 (71.22%)	14 (4.91%)	9 (3.15%)	12 (4.21%)	47 (16.49%)	10	
	**Percentage**	**0.0%**	**62.3%**	**4.3%**	**2.0%**	**3.0%**	**10.8%**	**17.5%**	
	**Total**	**0**	**374**	**26**	**12**	**18**	**65**	**105**	
What % of all inpatient non-medication orders are processed electronically?	Large (> = 400 beds)	0	63 (66.31%)	5 (5.26%)	4 (4.21%)	4 (4.21%)	19 (20%)	5	**0.586**
	Medium (100–399 beds)	0	125 (62.18%)	17 (8.45%)	10 (4.97%)	11 (5.47%)	38 (18.90%)	4	
	Small (6–99 beds)	0	180 (61.22%)	33 (11.22%)	19 (6.46%)	20 (6.80%)	42 (14.28%)	1	
	**Percentage**	**0.0%**	**61.3%**	**9.2%**	**5.5%**	**5.8%**	**16.5%**	**1.7%**	
	**Total**	**0**	**368**	**55**	**33**	**35**	**99**	**10**	

#### Clinical decision support

Table 10 presents the results regarding hospitals’ access to CDSS. CDSS was used in 69% of physician/nursing documents, 71.7% of medication orders, and 57.3% of non-medication orders. Additionally, although there is no significant relationship between hospital size and the use of CDSS in clinical documents and non-medication orders, the use of CDSS in medication orders has a significant relationship with hospital size. Small hospitals are better than medium-sized and larger hospitals in adopting CDSS for medication orders. Considering Tables 5, 9, and 10 together, we can infer that despite the fact that nearly all hospitals have pharmacy management systems and drug databases, small hospitals are adopting e-order and CDSS for medications more quickly than larger hospitals.

**Table 10:** Usage of CDSS

Questions	Hospital Size	Yes	No	Not Applicable	Missing	P
Clinical Documentation (Physician / Nursing Documentation)	Large (> = 400 beds)	63 (65.6%)	6 (6.3%)	27 (28.1%)	4	***p*** **< 0.001**
	Medium (100–399 beds)	131 (64.5%)	23 (11.3%)	49 (24.1%)	2	
	Small (6–99 beds)	220 (75.9%)	37 (12.8%)	33 (11.4%)	5	
	**Percentage**	**69.0%**	**11.0%**	**18.2%**	**1.8%**	
	**Total**	**414**	**66**	**109**	**11**	
Medication Orders	Large (> = 400 beds)	64 (66.0%)	4 (4.1%)	29 (29.9%)	6	**0.087**
	Medium (100–399 beds)	144 (71.6%)	9 (4.5%)	48 (23.8%)	4	
	Small (6–99 beds)	225 (76.5%)	18 (6.1%)	51 (17.3%)	1	
	**Percentage**	**71.7%**	**5.2%**	**21.3%**	**1.8%**	
	**Total**	**430**	**31**	**128**	**11**	
Non-Medication Orders	Large (> = 400 beds)	48 (85.7%)	7 (12.5%)	1 (1.8%)	44	***p*** **< 0.001**
	Medium (100–399 beds)	114 (72.2%)	43 (27.2%)	1 (0.6%)	47	
	Small (6–99 beds)	182 (65.7%)	71 (25.6%)	24 (8.7%)	18	
	**Percentage**	**57.3%**	**20.2%**	**4.3%**	**18.2%**	
	**Total**	**344**	**121**	**26**	**109**	

#### Closed-loop medication administration

Table 11 shows that 69.2% of hospitals have a second line of validation for prescriptions conducted by pharmacists before the drug is delivered to the wards and patients. On the other hand, only 0.8% of hospitals have an automatic dispensing system for drugs which means that drugs are delivered from pharmacies to the wards and then from the ward station to the bedside by the staff via trolleys/carts. This method is acceptable for drug safety, even if it is not very time and cost-efficient when compared to automated medication dispensing (AMD) systems [67, 68].

**Table 11:** Usage of CLMA

Questions	Hospital Size	Yes	No	Not Applicable	Missing	P
2nd line of validation for medication prescriptions which is documented electronically	Large (> = 400 beds)	53 (88.3%)	7 (11.7%)	0 (**0.0%)**	40	**0.271**
	Medium (100–399 beds)	132 (80.5%)	32 (19.5%)	0 (**0.0%)**	41	
	Small (6–99 beds)	230 (79.3%)	60 (20.7%)	0 (**0.0%)**	5	
	**Percentage**	**69.2%**	**16.5%**	**0.0%**	**14.3%**	
	**Total**	**415**	**99**	**0**	**86**	
Automated Dispensing of medication is available	Large (> = 400 beds)	3 (5.0%)	57 (95.0%)	0 (**0.0%)**	40	0.022*
	Medium (100–399 beds)	1 (0.6%)	160 (99.4%)	0 (**0.0%)**	44	
	Small (6–99 beds)	1 (0.3%)	291 (99.7%)	0 (**0.0%)**	3	
	**Percentage**	**0.8%**	**84.7%**	**0.0%**	**14.5%**	
	**Total**	**5**	**508**	**0**	**87**	
Closed-loop medication administration at the point of care	Large (> = 400 beds)	60 (75.9%)	19 (24.1%)	0 (**0.0%)**	21	***p*** **< 0.001**
	Medium (100–399 beds)	117 (62.9)	69 (37.1%)	0 (**0.0%)**	19	
	Small (6–99 beds)	93 (31.8%)	199 (68.2%)	0 (**0.0%)**	2	
	**Percentage**	**45.0%**	**47.8%**	**0.0%**	**7.0%**	
	**Total**	**270**	**287**	**0**	**42**	
Electronic Medication Administration Record (EMAR) available at point of care/bedside?	Large (> = 400 beds)	44 (44.0%)	18 (18.0%)	38 (38.0%)	0	***p*** **< 0.001**
	Medium (100–399 beds)	99 (48.8%)	65 (32.0%)	39 (19.2%)	2	
	Small (6–99 beds)	105 (36.6%)	181 (63.1%)	1 (0.3%)	7	
	**Percentage**	**41.3%**	**44.0%**	**13.0%**	**1.5%**	
	**Total**	**248**	**264**	**78**	**9**	

*p < 0.05

Furthermore, the survey suggests that only 45% of hospitals use technology at the bedside when administering medicines *(*i.e.*, barcode and Radio-frequency identification (RFID))* to electronically confirm the Institute for Healthcare Improvement’s Five Rights of Medication Administration: right patient, right drug, right dose, right time and right path [69, 70]. Similarly, only 41.3% of hospitals are immediately recording drug administration at the bedside, which means that nurses are recording the drug administration at the ward station after leaving the patient’s side. Interestingly, there is no significant relationship between hospital size and the use of CLMA functions, with the exception of “second line validation for medical prescriptions, which is documented electronically.” Table 11 indicates that small hospitals are worse than larger hospitals at providing second line validation of prescriptions electronically. To explain this result, we hypothesize that larger hospitals may have more financial and human resources to implement second-line validation.

Table 12 shows the items or persons (i.e.*, patient* and *nurse*) to be identified using technologies such as RFID or barcodes during medication administration at bedside. Our results show that technology is used more frequently to identify medications and patients than to identify nurses. While these percentages are higher in large and medium hospitals, they are significantly lower in small hospitals. Moreover, the *p*-value indicates that there is a significant relationship between the auto-identified target (medication, nurse, and patient) and the use of technology. This result implies that nurses do not consider it a necessity to validate themselves and their patients electronically but do validate medication administration at bedside using technology.

**Table 12:** Usage of The Five Rights of Medication Administration at bedside

Questions	Hospital Size	Auto-identified	Yes	No	Missing	P
Which of the following is auto-identified during bedside medication administration?	Large (> = 400 beds)	Medication	58 (69.0%)	26 (31.0%)	16	0.028*
		Nurse	43 (51.2%)	41 (48.8%)	16	
		Patient	57 (67.9%)	27 (31.1%)	16	
		**Percentage**	**52.7%**	**31.3%**	**16.0%**	
		**Total**	**158**	**94**	**48**	
	Medium (100–399 beds)	Medication	124 (61.7%)	77 (38.3%)	4	0.003**
		Nurse	95 (47.3%)	106 (52.7%)	4	
		Patient	125 (62.2%)	76 (37.8%)	4	
		**Percentage**	**55.9%**	**42.1%**	**2.0%**	
		**Total**	**344**	**259**	**12**	
	Small (6–99 beds)	Medication	104 (35.5%)	189 (64.5%)	2	**0.422**
		Nurse	99 (33.8%)	194 (66.2%)	2	
		Patient	114 (38.9%)	179 (61.1%)	2	
		**Percentage**	**35.8%**	**63.5%**	**0.7%**	
		**Total**	**317**	**562**	**6**	

*p < 0.05, **p < 0.01

#### Image management

IMS are stand-alone applications that are integrated with EHRs or HIS for practical usage. Table 13 shows that 74.7% of hospitals integrated their IMS with EHRs. When we consider the prevalence of IMS in hospitals, we can see that 37.3% of hospitals are using IMS in greater than 50% of the entire facility. There is no significant relationship between hospital size and the percentage of IMS integration with EHRs or with the prevalence of IMS. This situation can be explained by a nation-wide teleradiology system implemented by the Turkish MoH since 2008 [47].

**Table 13:** IMS Integration with EMR

Question	Hospital Size	Yes	No	Not Applicable	Missing	P
Is your IMS solution integrated with your Electronic Medical Record (EMR)?	Large (> = 400 beds)	57 (95.0%)	1 (1.7%)	2 (3.3%)	40	***p*** **< 0.001**
	Medium (100–399 beds)	155 (95.7%)	4 (2.5%)	3 (1.9%)	43	
	Small (6–99 beds)	236 (81.7%)	10 (3.5%)	43 (14.9%)	6	
	**Percentage**	**74.7%**	**2.5%**	**8.0%**	**14.8%**	
	**Total**	**448**	**15**	**48**	**89**

**Table 14:** Prevalence of IMS

Question	Hospital Size	100% (all)	76–100%	51–75%	26–50%	1–25%	Not Applicable	Missing	p
What % of medical images in all other departments are managed by your IMS?	Large (> = 400 beds)	12 (20.7%)	19 (32.8%)	9 (15.5%)	3 (5.2%)	7 (12.1%)	8 (13.8%)	42	***p*** **< 0.001**
	Medium (100–399 beds)	24 (14.8%)	46 (28.4%)	20 (12.3%)	11 (6.8%)	15 (9.3%)	46 (28.4%)	43	
	Small (6–99 beds)	35 (12.3%)	40 (14.0%)	19 (6.7%)	21 (7.4%)	23 (8.1%)	147 (51.6%)	10	
	**Percentage**	**11.8%**	**17.5%**	**8.0%**	**5.8%**	**7.5%**	**33.5%**	**15.8%**	
	**Total**	**71**	**105**	**48**	**35**	**45**	**201**	**95**	
What % of medical images in Radiology are managed by your IMS?	Large (> = 400 beds)	29 (48.3%)	26 (43.3%)	2 (3.3%)	1 (1.7%)	0 (0.0%)	2 (3.3%)	40	***p*** **< 0.001**
	Medium (100–399 beds)	57 (34.8%)	89 (54.3%)	11 (6.7%)	1 (0.6%)	3 (1.8%)	3 (1.8%)	41	
	Small (6–99 beds)	133 (45.5%)	71 (24.3%)	24 (8.2%)	10 (3.4%)	11 (3.8%)	43 (14.7%)	3	
	**Percentage**	**36.5%**	**31.0%**	**6.2%**	**2.0%**	**2.3%**	**8.0%**	**14.0%**	
	**Total**	**219**	**186**	**37**	**12**	**14**	**48**	**84**	

## Discussion

This study shows that HIS and main ancillaries, such as laboratory, radiology, and pharmacy information systems, are present in all Turkish hospitals included in this study. The availability of LIS, PAS, PACS, and MIS varies between 63.6 and 94%. The prevalence of EHR functions such as CPOE, CDSS, clinical documents, and drug management ranges from 70 to 99.5%. The size of the hospital has no significant relationship with the availability of those applications.

On the other hand, results show that not all hospitals that have such applications have adopted them. The proportion of hospitals that have adopted such applications and functions in at least half of the hospital varies between 57.3 and 79.3%. Despite the relationship between hospital size and availability of the applications, there is a significant relationship between the hospital size and the adoption of some EHR applications or functions. For example, the adoption of clinical documents, medication, and non-medication orders is higher among small hospitals compared to larger hospitals. Similarly, the use of CDSS during drug orders is more frequently adopted by small hospitals. Larger hospitals perform better than smaller hospitals only with regard to second-line verification of medication administration at bedside, which may be explained by available resources, i.e., nurses.

Jha et al. [22], DesRoches et al. [71], and Hikmet et al. [72] showed that small hospitals in the USA are significantly slower to adopt EHR functions than larger hospitals. One reason may be extracted from the study of Thakkar and Davis, which posed that the perception of hospital managers in the USA is that the benefits of EHR adoption are greater in larger hospitals than in smaller ones [73]. In addition, large hospitals in the USA may have larger financial and human resources to dedicate to the adoption of EHR functions than smaller hospitals might have. Turkey, as a centralized state, may be smaller than the USA, but the MoH can actively regulate all state hospitals nation-wide. Small hospitals in Turkey may use their size as an advantage to change more quickly than large hospitals. Nevertheless, when we consider the second line validation for medication administration at bedside, larger hospitals in Turkey also perform better, most probably due to more available financial and human resources, as suggested by Thakkar and Davis [73].

When we consider the concepts of basic and comprehensive EHR functions as defined by Jha et al. [21], by comparing them with EMRAM stages (as expressed in Table 1), we found that 63% of all hospitals surveyed in Turkey have at least basic EHR functions, and 36% have comprehensive EHR functions. This result is better than the results of Korean hospitals in 2017 [29], but lower than that of hospitals in the USA in 2015 [24] and 2017 [25], as depicted in Table 15.

**Table 15:** Comparison EHR adoption of the USA (in 2017), Korea (in 2017) and Turkey (in 2014–17)

Hospital Size	USA	Korea	Turkey
Basic EHR functions	41.4%	NA	27.1%
Comprehensive EHR functions	39.1%	NA	36%
Hospitals having at least basic EHR functions	80.5%	58.1%	63.1%

Additionally, the Turkish experience summarized in this study strengthens the claim [34–36] that following a bottom-up approach to encouraging the adoption of EHRs in public hospitals employed in the USA, is more successful than the top-down approach used in the UK.

Measuring country-wide EHR adoption is becoming widespread in the literature. Not only developed and developing countries, low- and middle-income countries such as Kenya [74] and Ghana [75] are also measuring their EHR adoption level. There are several models used in those studies. The survey used by Jha et al. in 2009 [21] is the pioneer of many studies; the same survey has been used by other researchers like in Korea [29]. On the other hand, many countries, such as Japan [33], China [30], developed their maturity models. Finally, some countries such as Canada [55], Portugal [54], and Turkey, as in this study, preferred to use HIMSS EMRAM as a maturity model.

Besides country-wide studies, there is an increasing number of studies about EHR adoption in the health system. The most recent review published in 2019 criticized 18 studies between 2005 and 2017, which are applied in different healthcare facilities from primary to tertiary healthcare services [76].

## Conclusion

Measuring the national adoption rates of EHR functions provides critical information and insights for healthcare policymakers. Despite all practical difficulties, studies measuring the overall adoption level of EHRs are increasing in number. This study is the first one to cover all state hospitals in a country using the EMRAM model developed by HIMSS. As the first nation-wide study in Turkey, the results may enable researchers to compare among countries like the USA, Korea, and China. Contrary to the conclusions of previous studies [22, 71, 72], this study found that smaller hospitals are better at adopting most EHR functions, with the exception of second-line validation for medication administration at bedside.

Additionally, as in the USA [34–36], this study found that after all required standardization and infrastructural studies, applying a bottom-up approach to adopting EHR functions in state hospitals has been successful in Turkey.

The results of this study are used by the MoH of Turkey to disseminate the benefits of EHR functions across the country. In consideration of studies showing the effect of using EHR functions on increasing healthcare quality, the Turkish MoH’s experience of using EMRAM may suggest that measuring adoption rates of EHR functions can be a good starting point for a healthcare authority to set targets to improve healthcare quality.

For further studies, it may be interesting to focus on measuring the correlation of EHR adoption level and healthcare quality scores measured by international standards, such as Joint Commission International, etc.

## Data Availability

The datasets generated and analyzed during the current study are not publicly available due to MoH regulations but are available from the corresponding author upon reasonable request.

## Abbreviations

AMD: Automated Medication Dispensing
CDR: Clinical Data Repository
CDSS: Clinical Decision Support Systems
CPOE: Computerized Physician Order Entries
EHR: Electronic Health Record
eMAR: Electronic Medication Administration Records
EMB: Electronic Medical Billing
EMRAM: Electronic Medical Record Maturity Model
EO: Electronic Ordering
HIE: Health Information Exchange
HIMSS: Healthcare Information and Management Systems Society
HIS: Hospital Information System
HL7: Health Level Seven International
ICD-10: International Classification of Diseases, 10th Revision
IMS: Image Management Systems
IT: Information Technology
MEG: Model of EHR Grading
MHLW: Ministry of Health, Labour and Welfare
MoH: Ministry of Health
MS: Medication Safety
NHDD: National Health Data Dictionary
PACS: Picture Archiving and Communication Systems
PAS: Patient Administration Systems
PD: Physician Documents
RFID: Radio-Frequency Identification
SA: Software Applications
SKS: Turkish national healthcare quality standards
SU: Software Usage
UK: United Kingdom
US: United States
USA: United States of America
